# An Investigation of the Risk Factors Related to Frailty in Older Adults Receiving Home Care Services

**DOI:** 10.3390/nu16233982

**Published:** 2024-11-21

**Authors:** Eirini Stratidaki, Enkeleint A. Mechili, Christina Ouzouni, Athina E. Patelarou, Ioannis Savvakis, Konstantinos Giakoumidakis, Aggelos Laliotis, Evridiki Patelarou

**Affiliations:** 1 Department of Nursing, School of Health Sciences, Hellenic Mediterranean University, 71004 Heraklion, Crete, Greece; ddk74@edu.hmu.gr (E.S.); apatelarou@hmu.gr (A.E.P.); savvakis.pt@gmail.com (I.S.); kongiakoumidakis@hmu.gr (K.G.); 2 Department of Healthcare, Faculty of Health, University of Vlora, 9401 Vlora, Albania; mechili@univlora.edu.al; 3 School of Medicine, University of Crete, 71500 Heraklion, Crete, Greece; 4 Occupational Therapy Department, University of West Attica, 12243 Egaleo, Greece; ouzouni@uniwa.gr; 5 Department of General Surgery, Venizeleio General Hospital, 71409 Heraklion, Crete, Greece; laliotisac@gmail.com

**Keywords:** frailty, home care, older adults, quality of life

## Abstract

(1) Background: Frailty in older adults is a condition that involves an interaction of psychological, biological, and social factors. This study aimed to assess the frailty status of older adults (65 years old and above) who receive home care services. Additionally, this work aimed to explore the key factors that have a statistically significant impact on the frailty of this vulnerable population. (2) Methods: This study represents the first phase of an intervention trial involving individuals aged 65 and over who received primary healthcare services and resided in the municipality of Archanes-Asterousia in Crete, Greece. Frailty was assessed using the SHARE-Frailty Instrument, while nutritional status was evaluated with the Mini Nutritional Assessment. Diet-related factors were analyzed, including health factors (oral hygiene, depression, cognitive decline, impaired functioning, quality of life), social factors (educational attainment, marital status, type of work before the age of 60), and lifestyle factors (smoking, alcohol consumption, diet). (3) Results: A total of 730 older adults were evaluated (31.5% male), with an average age (±SD) of 76.83 (±6.68) years. The frailty status analysis revealed 108 (14.8%) to be frail, 249 (34.1%) to be pre-frail, and 373 (51.1%) to be non-frail. Statistically significant associations were found between the MNA and Barthel scores (rs = 0.822, *p* < 0.001). Higher nutritional evaluations (MNA) were revealed in non-frail adults (mean (±SD); 26.97 ± 1.96) compared to pre-fail (mean (±SD); 19.37 ± 3.36) and frail adults (mean (±SD); 13.08 ± 3.16), as well as in pre-fail compared to frail adults (F = 1338.08, *p* < 0.001). Functional independence (Barthel) significantly differed with the frailty status of older adults (H = 521.98, *p* < 0.001; median for non-frail: 20.00, pre-fail: 19.00, frail adults: 15.00). (4) Conclusions: This study demonstrated that good nutritional status, good oral health, functional independence, and good quality of life are strongly correlated with lower frailty. Additionally, having chronic conditions is positively associated with one’s frailty status. Educational programs for both healthcare personnel and older adults are recommended.

## 1. Introduction

Frailty is a complex clinical syndrome characterized by reduced biological resilience and increased vulnerability in older adults to both internal and external stressors. Often associated with aging, frailty arises from multiple biological, psychological, and social factors that collectively reduce functional reserve. It is distinct from normal aging, as it involves the accumulation of pathological changes that impair the body’s ability to recover from injuries or illnesses. Frailty is defined by the presence of at least three of five key clinical features: weakness (decreased grip strength), slowness (reduced walking speed), weight loss, low physical activity, and exhaustion. Individuals meeting one or two of these criteria are classified as “pre-frail”, while those who meet none are considered “non-frail” [1,2].

Frailty is regarded as an early stage of disability and, as such, can often be mitigated or even reversed through timely and appropriate interventions, which may prevent, delay, or potentially reverse its progression [3]. Research shows that frailty is associated with older age, male gender, comorbidity, cognitive decline, depression, and a higher frequency of healthcare utilization [4,5,6]. The prevalence of frailty in the community ranges from 3.9% to 51.4% of the population, depending on the geographic area and assessment tools used in each country. In Greece, the prevalence of frailty among individuals aged 65 years and older was found to be 14.7%, with an additional 44.9% identified as pre-frail, according to the criteria of the SHARE study [7,8].

Recent studies have shown a correlation between weakness and nutritional status [9]. Aging anorexia is generally defined as a loss of appetite or reduced food intake and is considered a modifiable risk factor for frailty [9,10,11]. Weight loss is associated with a decrease in lean mass specific to skeletal muscle mass, reducing energy production for both aerobic and anaerobic metabolism. Weight loss leads to lower muscle strength, poor physical performance, a feeling of exhaustion, and, therefore, reduced physical activity [9,12,13].

Undernutrition is a well-recognized factor contributing to frailty, as it has been linked to an increased risk of developing frailty. This clearly demonstrates that inadequate nutrition and a low protein intake have been associated with a greater risk [14]. Inadequate nutrition has also been associated with many health conditions, including an increased risk of chronic diseases, decreased antioxidant defenses, a decreased immune response, an increased risk of osteoporotic fractures, and peripheral artery disease leading to vulnerability [15].

Oral health issues, such as pain and abscesses, can lead to difficulties in eating and chewing, which significantly affect the nutritional status of older adults [16]. Therefore, older people who wear dentures need to be taught proper home care of both the dentures and the tissues on which they rest, as well as the need for regular and ongoing professional care. In older adults, caloric needs generally decrease because of a lower basal metabolic rate, which results from a decline in muscle mass and reduced physical activity levels. However, certain groups of older people, such as those at home, without access to sunlight, may be deficient in vitamin D, too, and develop an increased risk of falls [17].

Frailty syndrome is considered reversible in its early stages and can be managed and prevented. For example, a systematic review focusing on primary care interventions highlighted that frailty is potentially reversible with timely interventions such as exercise, nutrition, and multifaceted healthcare support [18,19,20]. In longitudinal studies, frailty has also been found to be a risk factor for mortality, disability, and hospitalization [2,21]. Therefore, a better understanding of the factors involved in its development is of the outmost importance [22]. Despite all these data, further research is needed to characterize the frailty phenotype [21,23]. Few studies have examined the factors impacting the frailty condition of older adults in Greece, and almost no research has been conducted in the study area to date, where the very high life expectancy and characteristic lifestyle (psychosocial factors, sun exposure, food consumption, and physical activity in open spaces) may affect the frequency and variables related to frailty. A recent study conducted by the authors focused on an assessment of nutritional risk and the key factors among older adults in Greece [24].

Therefore, we hypothesized that socioeconomic factors—including limited education, living alone, low income, gender, type of residence, employment status, lifestyle, level of independence, cognitive impairment, depression, and nutritional status—might be linked to an increased risk of frailty in older adults. The general aim of this study included assessing the frailty status of the older adult population (aged 65 and older) receiving home care services in Asterousia, Heraklion Prefecture, Crete, Greece, from the “Home Help” program and the Open Care Center for Older adults. The specific objectives were as follows:

- Assessing the key factors that have a statistically significant impact on frailty in this vulnerable population;
- Mapping the status among older adult individuals;
- Identifying the key determinants of the risk of frailty among older adults.

## 2. Materials and Methods

This study was the first phase of an intervention trial that took place between August 2021 and July 2023 in the Municipality of Archanes-Asterousia, Heraklion Prefecture, Crete. The sample consisted of 730 individuals over the age of 65 who were members of the Open Protection Centre for Older adults and “Help at Home” programs. Data collection was performed through face-to-face appointments scheduled with the older adults at the center and during home visits. During these visits, the necessary information was obtained through questionnaires, and somatometric measurements were made. The somatometric measurements included assessments of frailty, nutritional status, oral health, the level of functionality and independence, cognitive impairment, geriatric depression, the incidence of comorbidities, the extent of homebound status, and quality of life.

Briefly, the scales used for the purposes of the study were the Greek version of the Mini Nutritional Assessment (MNA) tool [25], the Oral Health Assessment Tool (OHAT) [26], the Barthel Index for Activities of Daily Living [27], the Mini Mental State Examination (MMSE) [28], the Greek version of the Geriatric Depression Scale (GDS) [29], the Charlson Comorbidity Index (CCI) [30], the extent of homebound status [31], and the Greek version of the WHOQOL-BREF scale [32].

Moreover, frailty was assessed using the five frailty phenotype indicators described by Fried et al. [2]: (1) unintentional weight loss: defined as a weight loss of more than or equal to 4.5 kg, or greater than or equal to 5% of body weight, assessed through self-report; (2) self-reported exhaustion; (3) gait speed: measured by timing, in seconds, how long it took to walk 4.6 m, with cut-off points determined based on the 80th percentile of the walking time, adjusted for gender and height (in meters); (4) grip strength: evaluated using a handgrip strength test with a portable hydraulic hand dynamometer (Saehan^®^, Changwon-si, Republic of Korea), adjusted for gender and body mass index (BMI); and (5) the physical activity level. Participants were classified as non-frail (meeting none of the frailty criteria), pre-frail (meeting one or two criteria), or frail (meeting three or more criteria), according to the defined thresholds [33].

The methodology has been described in detail elsewhere [24].

### 2.1. Statistical Analysis

After data collection, the survey responses were entered into and analyzed using SPSS 25.0 (IBM Statistical Package for Social Sciences for Windows, Version 25.0. Armonk, NY, USA: IBM Corp). A 5% level of statistical significance was set.

The qualitative variables in the study were described using both absolute (*n*) and relative frequencies (%). For quantitative variables that met the assumption of normality, the mean (M) and standard deviation (SD) are reported; for those that did not, the median and interquartile range (IQR) were used. The normality of variables was assessed through the kurtosis and skewness values.

Pearson’s correlation coefficient (r) was used to examine associations when variables met the normality assumption, while Spearman’s rho was applied otherwise.

The χ^2^ test assessed the independence between categorical variables. For mean and median comparisons across independent samples, the *t*-test and Mann–Whitney U test were applied, based on whether the normality assumption held.

To examine differences across more than two categories, such as in the analysis of vulnerability, an analysis of variance (ANOVA) was used along with the post hoc Games–Howell test when normality was satisfied; otherwise, the non-parametric Kruskal–Wallis H test was employed. For the logistic regression model, the “Enter” method was used to input variables.

### 2.2. Ethical Approval and Consent to Participate

This study adhered to the principles of the Declaration of Helsinki and received approval from the Research Ethics Committee of the Hellenic Mediterranean University (ID number: 47/7 January 2021). All regulations concerning individual protection and personal data processing were strictly followed, in compliance with the General Data Protection Regulation (GDPR) and the Data Protection Act of 2019.

## 3. Results

### 3.1. Demographic Profile of Participants

**Figure 1:**
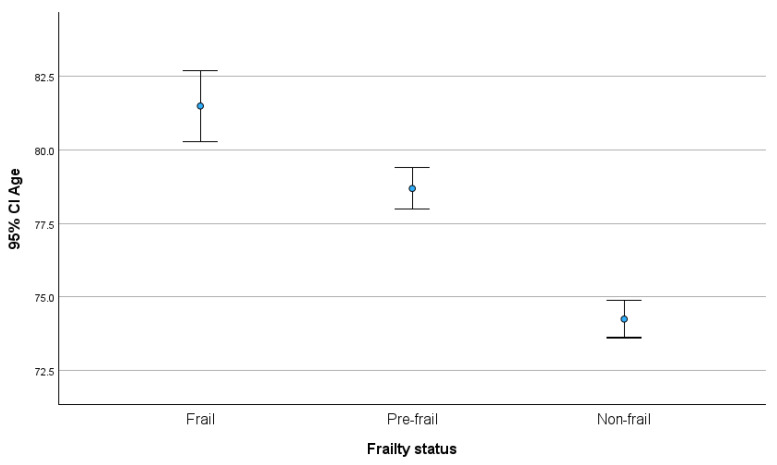
Age according to frailty status.

A total of 730 older people aged 65–96 years (mean = 76.83, standard deviation (SD) = 6.68) were included in this study. Their age differed according to their frailty status, with older participants more frequently presenting frailty (Figure 1). In all, 68.5% of the sample were female, and 97.9% were of Greek origin. Among the participants, 40.8% lived alone, while 59.2% reported cohabitating with another person; 7.9% had had a fall in the last month; while 9.7% were confined to their home, 28.9% were semi-confined, and 61.4% were not confined. Their demographic characteristics are presented in detail elsewhere [24].

The frailty status analysis revealed 14.8% (108) to be frail, 34.1% (249) to be pre-frail, and 51.1% (373) to be non-frail. The frail patients were older, had a worse financial status, were more often widowed/divorced or unmarried, lived alone, had had falls in the last month, and were homebound (Table 1).

### 3.2. Factors Linked to Frailty in Older Adults

Analyses were performed to explore the relationship between frailty and factors such as nutritional status, oral health, mental health, comorbidities, functional independence, and quality of life.

Statistically significant differences were found in all variables under examination (*p* < 0.001); more specifically, differences were found in all stages of vulnerability, i.e., between all categories.

More specifically, in the Charlson Comorbidity Index (CCI), higher scores were observed among the vulnerable (M ± SD = 6.54 ± 1.42) compared to both the pre-vulnerable (M ± SD = 5.18 ± 1.51) and the non-vulnerable (M ± SD = 4.37 ± 1.60). The differences between the pre-vulnerable and non-vulnerable were also statistically significant.

Inverse conclusions emerged from the examination of the remaining variables. More specifically, the nutritional status assessment (MNA), the oral hygiene assessment (OHAT), the Barthel functional independence scale, and all scales of the quality-of-life questionnaire (WHOQOL-BREF) showed higher scores in the group of non-frail participants compared to both the pre-frail and vulnerable. The pre-vulnerable showed higher levels compared to the vulnerable. For example, higher scores were observed in the nutrition status assessment (MNA) for the non-vulnerable (M ± SD = 26.97 ± 1.96) compared to both the pre-vulnerable (M ± SD = 19.37 ± 3.36) and the vulnerable (M ± SD = 13.08 ± 3.16). The differences between the pre-vulnerable and the vulnerable were also statistically significant (Table 2).

To investigate the impact of nutritional status, oral hygiene, depression, mental health, comorbidities, functional independence, and quality of life on vulnerability, logistic regression models were utilized. In this analysis, the independent variables included nutritional status, oral hygiene, depression, mental health, comorbidities, functional independence, and quality of life. Due to their collinearity, the subscales of WHOQOL-BREF were excluded from this logistic regression. The model demonstrated statistical significance (*p* < 0.001), with a Nagelkerke R^2^ value of 0.913, indicating a strong fit. Specifically, the findings revealed that effective oral hygiene, functional independence, and cognitive impairment act as protective factors against vulnerability. In other words, these elements decrease the likelihood of developing vulnerability (Table 3).

## 4. Discussion

This study aimed to assess the frailty status of the older adult population (65 years and above) receiving home care services in Asterousia, Heraklion Prefecture, Crete, Greece, through the “Help at Home” program and the Open Care Center for Older Adults. Additionally, this work attempted to explore the key factors that have a statistically significant impact on the frailty of this vulnerable population. Mapping the situation and finding the key determinants of vulnerability gives healthcare personnel, family members, and health policymakers the appropriate evidence to provide the needed healthcare services and to organize home services accordingly.

Despite the very popular Mediterranean diet in Greece, the current study reports a significant percentage of frailty among older adults. A study conducted in five European countries reported different results, with the prevalence of frailty among the older population ranging from 0 in Austria to 13.7% in Portugal. Additionally, the reported pre-frailty prevalence was higher in Portugal (57.3%) and lower in Germany (37.1%). These results must be analyzed and interpreted in a specific way [34]. No special differences are observed between Greece and Portugal, which are both Mediterranean countries and have populations with similar lifestyles. However, huge differences are observed with Germany, Austria, Switzerland, and France. All these are central European countries, with different healthcare systems and lifestyles. Another possible explanation for these differences is the higher percentages of physical inactivity in southern countries in comparison to central European countries [35,36]. Similar results were also reported in another European study among 18 countries [37], wherein the frailty prevalence in Greece was reported at a level of 9.9%. This small difference is normal because our study included only people above 65 years old, while in the previous study, participants of age 50 or higher were included. Moreover, it is worth mentioning that the prevalences among those of age 65–74 and 75–84 were reported at levels of 7.0% and 16.8%, respectively [37]. In this age group, the results do not differ much, and this is a clear indicator of the high frailty prevalence in Greece. Another study among ten European countries, published in 2009, reported that the frailty prevalence in Greece among the population aged 65+ was 14.7% [7]. A study in Greece conducted during the pandemic period reported that 58.3% of the participants were identified as frail [38]. The differences here are mainly attributed to the different instruments used, different methodological approaches, the COVID-19 pandemic, and the location in which the study took place. Despite the possible differences and key reasons, we can clearly say that frailty is very frequent among older adults in Greece. No specific decrease in prevalence has been observed in at least the last 15 years. The economic crisis and the austerity measures that Greece undertook in the last decade had a huge impact on the healthcare system [39]. Additionally, the institutionalization levels of elderly people are lower in Greece in comparison to other central European countries. This may be another possible explanation for the higher prevalence. Despite all the above, aging of the population is a phenomenon that affects healthcare systems and their sustainability. Increasing the percentage of the GDP that is allocated to the healthcare system, increasing the awareness of frailty among healthcare personnel, and undertaking health policy initiatives in Greece are strongly recommended.

The current study showed that a higher frailty prevalence is associated with worse nutritional status, oral hygiene, quality of life, and Barthel functional independence scores. A Greek study confirmed that frailty is negatively connected with quality of life [40]: the higher the frailty, the lower the quality of life. Another Greek study conducted in a northern city reported similar results to the current one. The authors concluded that all the dimensions of quality of life have a statistically significant negative correlation with frailty [41]. Similar results were also reported in a study in Poland [42]. Despite the different instruments that different studies use to measure frailty and quality of life, the majority of them conclude that frailty affects the population’s quality of life, especially that of older adults. The difficulties they feel are also transmitted in the social, physical, and mental health dimensions that are part of the quality of life. An early diagnosis of frailty can improve the results and additionally meliorate the quality of life [41,42].

It is worth mentioning that it is still not clear as to whether frailty leads to a worse quality of life or vice versa. However, regardless of the direction, it is clear that an improvement in one indicator also improves the other. A statistically significant correlation has been found in several studies between frailty and nutritional status in elderly people [43,44]. Older adults are often malnourished, and this can lead to frailty. A study reported that an improvement of 1 point in the MNA score decreases frailty by 13.8% [44]. It is important to improve both the quality (nutrient quality) and the quantity (energy intake) of nutrition for older adults in order to decrease their frailty [45]. Moreover, a recent review study concluded that a combination of nutritional therapy and exercise is the best approach for a positive result [46].

A systematic review reported a strong connection between oral health and frailty [47]. These results are in line with those of the current work and of a study conducted in Brazil [48], which showed that good oral health is a key determinant of vulnerability. Another systematic review emphasized that oral health could be used as an indicator of frailty [49]. Dental hygiene and the number of teeth are factors that can be used for an early diagnosis of frailty. Education of older adults on the importance of good oral health is strongly recommended. Additionally, the healthcare personnel that offer services to this vulnerable population need to be educated on how to teach older adults about oral health. Furthermore, good oral health could improve quality of life.

This study found that functional independence and frailty are strongly connected. This result has also been reported elsewhere [50,51]. Lower functional independence can lead to social isolation and mental health issues. Additionally, older adults with high dependence need someone to assist them in their daily activities. The implementation of different programs (i.e., home exercise rehabilitation programs) can decrease functional dependence [52]. Moreover, an improvement in nutritional status can decrease the dependence of the older adult population [43].

According to the results of the current work, people with comorbidities had a higher prevalence of frailty. Chronic conditions and frailty usually occur together and lead to a low quality of life and functional dependence [53]. People with chronic conditions are more vulnerable in comparison to those without. Additionally, chronic conditions deteriorate the quality of life, decrease functional independence, and often lead older adults to become malnourished. A holistic approach is needed to manage frailty, and specific strategies for home care services need to be developed and implemented. In challenging times, where funding for healthcare systems is crucial, improving home care services for the older adult population can lead not only to lower costs for healthcare systems but also to a healthier population.

### Strengths and Limitations

The current work had some limitations. This was a cross-sectional study, which caused difficulties in drawing conclusions regarding causality. Moreover, we collected data in only one setting, and this is a key limitation on the generalization of the results. However, the sample size of the current study, as well as the heterogeneity of the instruments used for data collection, provides a better overview of the status of older adults on the island of Crete. Future studies could increase the sample size and implement educational approaches for both healthcare personnel and older adults.

## 5. Conclusions

The prevalences of frailty, pre-frailty, and non-frailty were found to be at levels of 14.8%, 34.1%, and 51.1%, respectively, in a population 65+ years old that lives in Crete and receives services from the “Help at Home” program and the Open Protection Centre for Older Adults. Lower frailty was statistically connected with effective oral hygiene, functional independence, and cognitive impairment. Additionally, having a chronic condition is positively connected with one’s frailty status. Educational programs for both healthcare personnel and older adults are recommended. Frailty can be managed with holistic approaches. Additionally, the provision of home care services needs to be well designed and implemented.

## Figures and Tables

**Table 1:** Demographic and other characteristics categorized based on frailty status.

		Frail (*n* = 108)	Pre-Fail (*n* = 249)	Non-Frail (*n* = 373)	*p*-Value
Age (years, mean ± SD)		81.50 ± 6.34	78.69 ± 5.66	74.24 ± 6.25	**<0.001** ^‡^
Gender					0.288
	Male	33 (30.6%)	70 (28.1%)	127 (34.0%)	
	Female	75 (69.4%)	179 (71.9%)	246 (66.0%)	
Origin					0.136
	Greece	108 (100.0%)	245 (98.4%)	362 (97.1%)	
	Other	0 (0.0%)	4 (1.6%)	11 (2.9%)	
Annual individual income (EUR)					**0.043**
	>4500	66 (61.1%)	172 (69.1%)	274 (73.5%)	
	<4500	42 (38.9%)	77 (30.9%)	99 (26.5%)	
Annual family income (EUR)					0.278
	>9475	59 (57.8%)	139 (57.4%)	234 (63.4%)	
	<9475	43 (42.2%)	103 (42.6%)	135 (36.6%)	
Program					0.351
	Help at home	77 (71.3%)	195 (78.3%)	281 (75.3%)	
	Center for Open Protection of Older Adults	31 (28.7%)	54 (21.7%)	92 (24.7%)	
Smoker					0.145
	Current	18 (16.7%)	27 (10.8%)	61 (16.4%)	
	Ex	40 (37.0%)	121 (48.6%)	166 (44.5%)	
	Never	50 (6.3%)	101 (40.6%)	146 (39.1%)	
Educational level					0.189
	Lower	85 (78.7%)	171 (68.7%)	249 (66.8%)	
	Middle	21 (19.4%)	71 (28.5%)	116 (31.1%)	
	Higher	2 (1.9%)	7 (2.8%)	8 (2.1%)	
Knowledge of a foreign language					0.953
	None	89 (82.4%)	210 (84.3%)	313 (83.9%)	
	English	6 (5.6%)	10 (4.0%)	20 (5.4%)	
	German	8 (7.4%)	14 (5.6%)	22 (5.9%)	
	Other	5 (4.6%)	15 (6.0%)	18 (4.8%)	
Job					0.464
	Mental work	7 (6.5%)	20 (8.0%)	37 (9.9%)	
	Manual work	85 (78.7%)	197 (79.1%)	299 (80.2%)	
	Combination of mental and practical work	16 (14.8%)	32 (12.9%)	37 (9.9%)	
Family status					**<0.001**
	Unmarried	6 (5.6%)	8 (3.2%)	6 (1.6%)	
	Married	36 (33.3%)	139 (55.8%)	243 (65.1%)	
	Widowed/divorced	66 (61.1%)	102 (41.0%)	124 (33.2%)	
Do you live alone?					**<0.001**
	Yes	71 (65.7%)	106 (42.6%)	121 (32.4%)	
	No	37 (34.3%)	143 (57.4%)	252 (67.6%)	
Have you had falls in the last month?					**<0.001**
	No	63 (58.3%)	238 (95.6%)	371 (99.5%)	
	Yes	45 (41.7%)	11 (4.4%)	2 (0.5%)	
Homebound status					**<0.001**
	Confined	71 (65.7%)	0 (0.0%)	0 (0.0%)	
	Semi-confined	37 (34.3%)	174 (69.9%)	0 (0.0%)	
	Non-confined	0 (0.0%)	75 (30.1%)	373 (100.0%)	

Notes: Values refer to absolute and relative frequencies (%) or means ± standard deviations (SDs). *p*-values were computed using the chi-square test or ^‡^ *t*-test. Bold indications correspond to statistically significant findings.

**Table 2:** Investigation of levels of nutritional status (MNA), oral health (OHAT), mental status (MMSE), Charlson comorbidity (CCI), Barthel scale, and quality of life (WHOQOL-BREF) with regard to frailty.

		Frailty			*F*/*H* ^†^	*p*-Value
		Frail	Pre-Frail	Non-Frail		
Mini Nutrition Assessment (MNA)		13.08 ± 3.16	19.37 ± 3.36	26.97 ± 1.96	1338.084	**<0.001**
Oral Health Assessment Tool (OHAT)		8.32 ± 2.68	10.44 ± 2.58	12.69 ± 2.10	167.195	**<0.001**
Mini Mental State Examination (MMSE)		27.00 ± 1.95	27.44 ± 1.62	28.23 ± 1.51	32.475	**<0.001**
Charlson Comorbidity Index (CCI)		6.54 ± 1.42	5.18 ± 1.51	4.37 ± 1.60	86.237	**<0.001**
Charlson Comorbidity Index (CCI) [%] ^†^		0.00 (2.00)	21.00 (51.00)	53.00 (75.00)	37.135	**<0.001**
Barthel scale		15.00 (4.00)	19.00 (1.00)	20.00 (0.00)	521.981	**<0.001**
Barthel scale (0–100)		75.00 (20.00)	95.00 (5.00)	100.00 (0.00)	521.981	**<0.001**
Geriatric Depression Scale		8.53 ± 3.86	8.13 ± 3.59	7.99 ± 4.11	0.784	0.457
Quality of Life (WHOQOL-BREF)						
	Overall QOL/general health	9.13 ± 2.23	11.35 ± 2.55	14.34 ± 2.83	199.988	**<0.001**
	Physical health	9.96 ± 1.24	11.44 ± 1.63	13.80 ± 1.61	327.694	**<0.001**
	Psychological health	10.12 ± 1.63	11.90 ± 1.72	14.28 ± 1.65	319.358	**<0.001**
	Social relationships	8.36 ± 1.68	10.87 ± 2.26	15.17 ± 2.52	475.063	**<0.001**
	Environment	8.75 ± 1.61	11.21 ± 1.98	15.13 ± 2.00	586.778	**<0.001**

Notes: Values are mean ± standard deviation and Analysis of Variance (ANOVA) or ^†^ in median and interquartile range while control refers to Kruskal-Wallis H. Post-hoc analysis with Games-Howell test. Bold indications correspond to statistically significant findings.

**Table 3:** Multinomial logistic regression with frailty as the dependent variable.

	Model Fitting Criteria	Likelihood Ratio Tests		OR (95% CI) Pre-Frail	OR (95% CI) Frail vs. Non-Frail
	−2 Log Likelihood of Reduced Model	Chi-Square	*p*-Value		
Oral Health Assessment Tool (OHAT)	343.658	24.122	**<0.001**	0.693 (0.592, 0.812)	0.605 (0.479, 0.765)
Geriatric Depression Scale (GDS)	323.510	3.974	0.137	1.023 (0.929, 1.128)	0.891 (0.752, 1.057)
Mini Mental State Examination (MMSE)	344.804	25.268	**<0.001**	0.750 (0.578, 0.972)	1.737 (1.062, 2.841)
Charlson Comorbidity Index (CCI)	339.793	20.257	**<0.001**	1.036 (0.794, 1.351)	2.769 (1.567, 4.894)
Barthel Scale (0–100)	1071.436	751.900	**<0.001**	0.350 (0.298, 0.412)	0.222 (0.180, 0.274)

Notes: Bold indications correspond to statistically significant findings.

## Data Availability

The data presented in this study are available on request from the corresponding author.
